# The use of novel knotless barbed sutures in posterior long-segment lumbar surgery: a randomized controlled trial

**DOI:** 10.1186/s13018-022-03165-7

**Published:** 2022-05-18

**Authors:** Kai Shi, Xuanwei Chen, Bin Shen, Yue Luo, Renqin Lin, Yu Huang

**Affiliations:** 1 Department of Spinal Surgery, The First Affiliated Hospital of Fujian Medical University, No. 20 Chazhong Rd., Taijiang District, Fuzhou, 350005 Fujian Province China; 2 School of Foreign Languages, Fuzhou University, No. 2 Xueyuan Rd, University Town, Fuzhou, 350004 Fujian Province China; 3 Fujian Medical University, No. 88 Jiaotong Rd., Taijiang District, Fuzhou, 350005 Fujian Province China

**Keywords:** Barbed suture, Lumbar surgery, Wound closure, Randomized controlled trial

## Abstract

**Background:**

The study carries the aim to compare the clinical efficacy and economic outcomes of using barbed suture closure versus conventional closure for wounds after posterior long-segment lumbar surgery.

**Methods:**

One hundred and eighty-one patients undertaking posterior long-segment lumbar surgery participated in the prospective randomized controlled trial study to receive either barbed suture wound closure (*n* = 91) or conventional suture closure (*n* = 90). Outcome measures included operating room time (ORT), wound closure time, length of incision, length of hospital stay (LOS), 90-day readmission rates, wound complications of dehiscence and infection, and costs.

**Results:**

Barbed suture group was related with significantly lower ORT (*P* = 0.036), wound closure time (*P* < 0.001) and average wound closure time (*P* < 0.001), and significantly lower wound complication rates (dehiscence and infection) (*P* = 0.031). No significant differences were found when compared with conventional suture group in terms of length of incision (*P* = 0.086), length of hospital stay (*P* = 0.174), readmission rates up to 90 days after the surgical procedure (*P* = 0.232) and costs (*P* = 0.205).

**Conclusion:**

The study suggested the knotless barbed suture technique outperformed the conventional suture in shortening operating room time, wound closure time and average wound closure time, and reducing wound complication rates.

## Introduction

As a common surgical treatment for multi-level lumbar degenerative diseases, posterior long-segment lumbar surgery mainly refers to the surgery of three or more posterior segments suitable for lumbar disk herniation, lumbar spinal stenosis, lumbar spondylolisthesis and lumbar degenerative scoliosis/kyphosis. The application of accelerated recovery after surgery (ERAS) in this surgery is particularly critical, given distinctive surgery features of excessive amount of bleeding, long operation time, heavy destruction of paravertebral muscles and facets, high risk of potential dural damage and nerve injury, great operation cost, and high probability of postoperative complications, especially adjacent segmental disease (ASD).

Being an essential part of ERAS, successful wound closure plays an important role in achieving favorable postoperative outcomes of spinal surgery because it may pose effects on risk of surgical site infection (SSI), healing, post-acute care follow-up and patient self-care [1]. Recently, increasing attention has been fixed on the application of barbed sutures in enhancing wound closure with prior attempts made in a few surgical fields including cosmetology, obstetrics, tenorrhaphy, gynecology and orthopedic arthroplasty [2–4]. Previous findings have indicated that as compared with the use of traditional sutures, the use of barbed sutures is mostly characterized by less suturing time, faster wound closure, lower hospital costs, and fewer postoperative complications [5–13].

Barbed sutures are absorbable sutures incorporating barbs on the outer surface and can be anchored with the incision tissue. The tissue tension could be maintained without knotting after tightening the suture [14]. Nonetheless, conventional sutures are secured with interrupted knots, the process of which may result in higher rates of complications and be time-consuming [15].

In the past decades, while studies have been conducted on the comparison between tissue reaction scores of knotless barbed suture and conventional suture with knots, little evidence exists with regard to the clinical outcome, complications, cost and capacity of barbed sutures in spinal surgery. [16–18] To fill this void, this study aims to compare the clinical efficacy of using barbed suture closure versus traditional closure for wounds after posterior long-segment lumbar surgery.

## Methods

### Participants and randomization

From January 1^st^, 2020 to December 31^st^, 2020, a single-hospital prospective randomized controlled trial was conducted in the Department of Spine Surgery, the First Affiliated Hospital of Fujian Medical University. Our research team prospectively screened 252 patients who underwent a posterior long-segment lumbar surgery, including surgeries of three or more posterior segments of decompression/ internal fixation/ fusion/ correction of vertebral deformity (expansion, compression, de rotation, etc.), with or without osteotomy. Inclusion criteria were (1) 18 < age < 80 years old; (2) first time in posterior long-segment lumbar surgery; (3) be able to communicate well with researchers and follow the whole research protocol requirements. Exclusive criteria included (1) diabetic patients with poor glycemic control or with severe diabetic complications; (2) patients with long-term use of steroids; (3) patients with spinal tumors; (4) patients with scar constitution; (5) body-mass index (BMI) ⩾ 35 kg/m^2^. Among the 201 included patients, 20 declined to participate, with the most frequent reason being the fear and uncertainty toward the novel barb suture technique in the study group and they were therefore unable to be randomized. A total of 181 patients were enrolled in the study. Using a computerized randomization software, patients were equally divided to receive conventional sutures (control group) or barbed sutures (study group), resulting 91 in the study group and 90 in the control group. Participating surgeons and operating room personnel were, respectively, informed at the time of surgery. Outcome assessments were performed as usual at two weeks and 90 days postoperative to both groups of patients during regular clinic visits.

Baseline demographic data and medical history are collected and presented in Table 1. No statistically significant differences between the two groups were observed. (*p* > 0.05).

**Table 1:** Baseline patient demographics

		Barbed suture (N = 91)	Conventional suture (N = 90)	*t/χ*^2^	*P*
Age		55.85 ± 17.1	52.97 ± 19.92	1.041	0.299
Gender	Male	47	44	2.973	0.85
	Female	35	55		
Smoking status		20	12	2.221	0.136
BMI		23.21 ± 3.96	22.20 ± 4.14	1.579	0.116
Diagnosis	Spinal scoliosis	21	29	7.360	0.118
	Lumbar disk herniation	43	39		
	Lumbar spinal stenosis	17	13		
	Lumbar fracture	5	0		
	Lumbar spondylolisthesis	4	6		
	Others	1	3		

### Research procedures

General anesthesia was performed in all patients undergoing lumbar surgery in this study. All patients adopted the traditional posterior median approach to dissect the paraspinal musculature from the spinous processes and laminae in a subperiosteal manner. Whether lamina decompression is needed is contingent on various types of diseases. All patients were treated with pedicle screw internal fixation, and the suturing for both groups was completed by the same group of surgeons with qualified suturing technique.

#### Conventional suture technique

For the control group, sutures of three layers were used to perform wound closure. The closure of the fascial layer was achieved using interrupted absorbable suture size 0 Vicryl (Ethicon Inc.), followed by size 2-0 Vicryl (Ethicon Inc.) for the dermal layer and finally the subcuticular layer was sutured by 3-0 Vicryl (Rapide Inc.).

#### Barbed suture technique

**Fig. 1:**
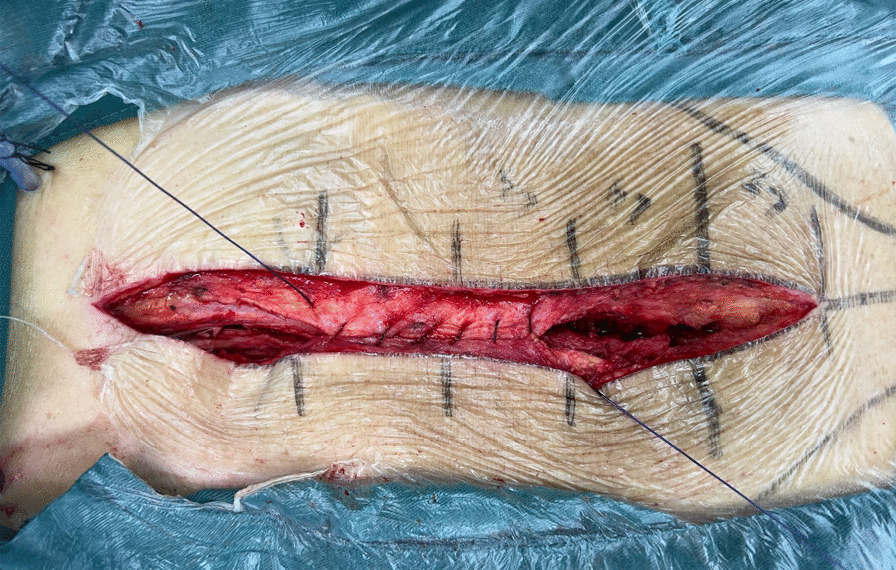
Image of fascia and dermal layer closure from the midpoint to both ends of the incision

**Fig. 2:**
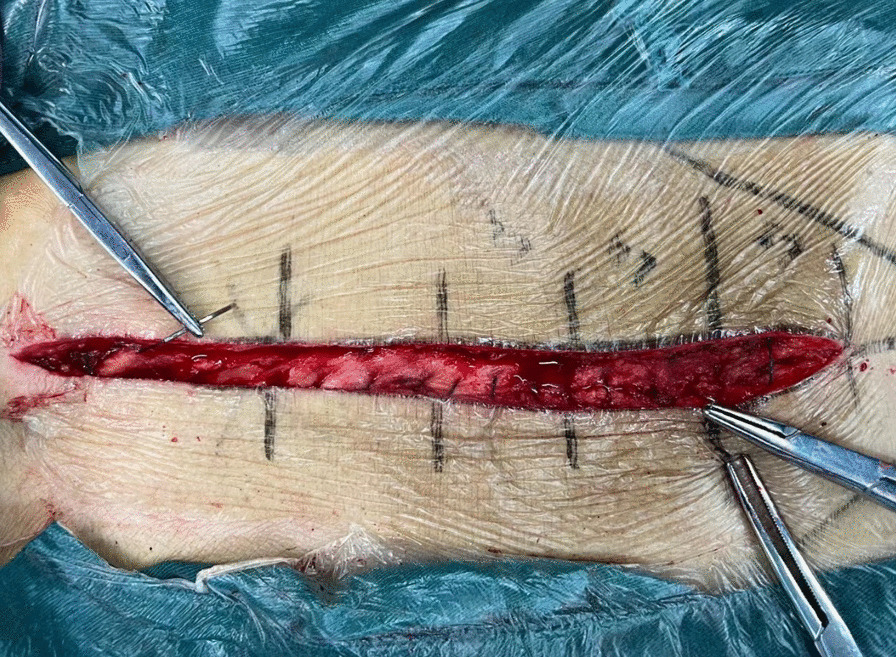
Image of backward suturing from both ends to the midpoint of the incision

**Fig. 3:**
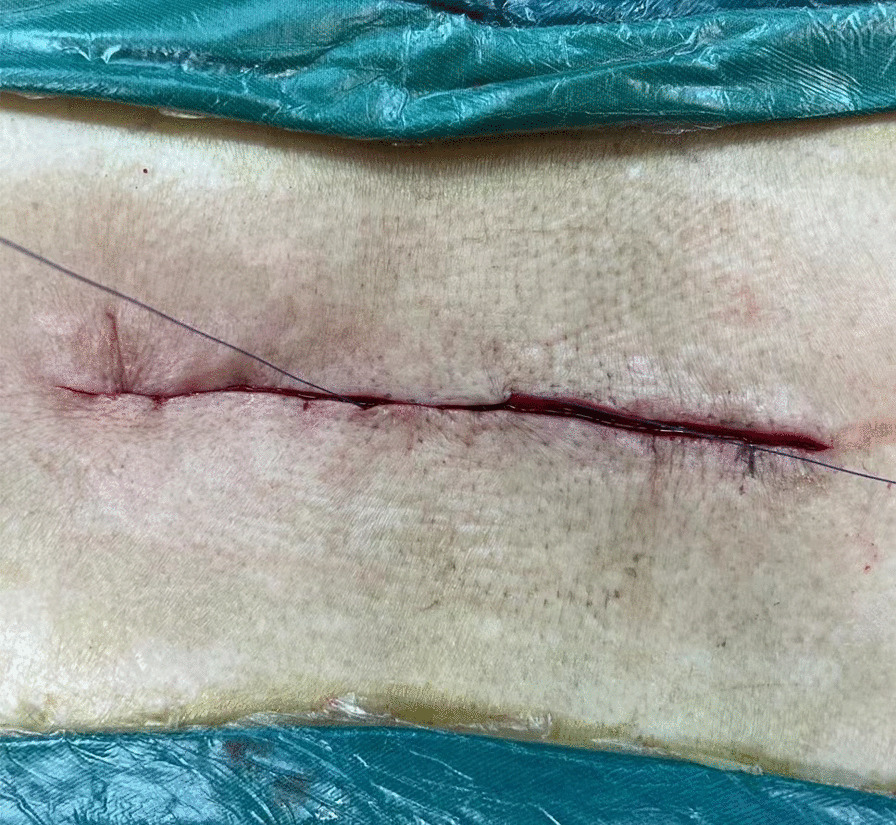
Image of subcuticular layer closure from the midpoint to both ends of the incision

**Fig. 4:**
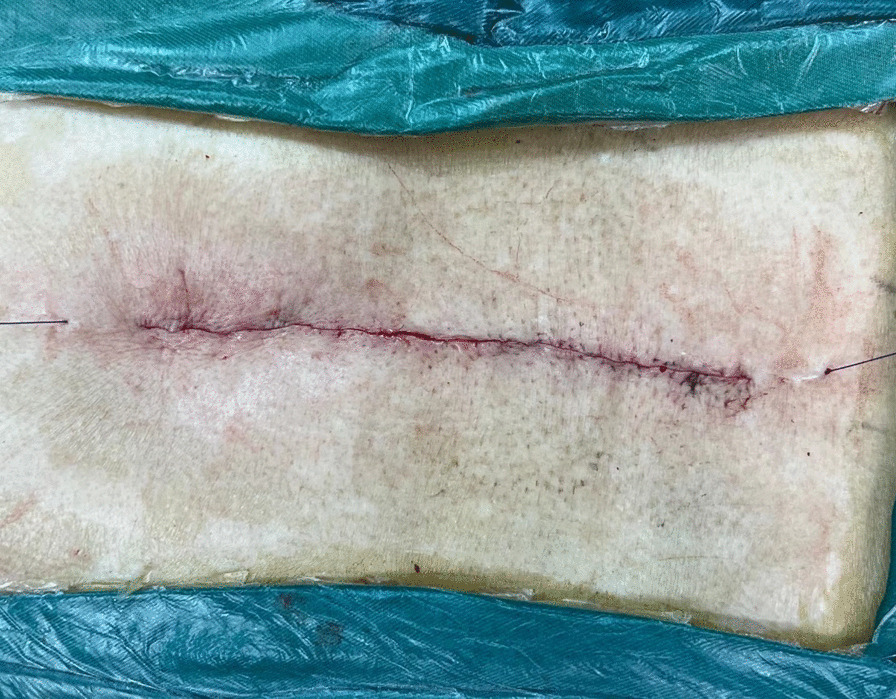
Image of continuing the suture beyond the end of the incision and exiting out

For the study group, suturing was performed with Fenkuill, a type of absorbable bidirectional barbed suture made by Hainan Jianke Pharmarceutical Co., LTD. The fascia was sutured with no interruptions with a size 2 Fenkuill; the dermal layer was sutured with size 2 or size 1 Fenkuill, and the subcuticular layer with a size 2-0 Fenkuill. For both fascia and dermal layers, the suture started from the middle of the incision and the suturing was conducted by two surgeons from the middle point to both ends of the incision at the same time, leaving 0.5–1 cm between every two suture throws (Fig. 1). When the end of the incision is reached, a backward suturing running from both end sides to midpoint of the wound was performed with one to two throws to further strengthen the suture. The rest of the suture was then cut at its exit point (Fig. 2). For the subcuticular layer, the suturing also began from the midpoint to both sides in a similar fashion, except that when reaching the end of the wound, the suturing continued beyond a bit and exited out at around 2 cm away and the extra suture was removed (Figs. 3 and 4). No surgical knots were placed in any of the barbed suture and the tension of soft tissue on both sides of the incision was kept balanced during the suturing process.

### Outcome measures

The following outcomes of both groups were measured:

- Operating room time (ORT)
- Wound closure time (from placement of the first stitch to the completion of wound closure)
- The length of incision
- The average suturing time (calculated by formula: the average suturing time = the suturing time/the length of incision)
- Length of hospital stay (LOS)
- 90-day readmission rates
- Wound complications (dehiscence and infection)
- Cost (the suture material cost and the total hospitalization expenses)

Readmission was defined as any event related to posterior long-segment lumbar surgery wound condition that may require readmission to the same hospital. Wound complications were defined as any wound-related event that may require a reoperation or change in perioperative care. In this study, wound dehiscence and infection were measured as manifestations of wound infections, where the former was defined as failure of wound closure leading to compromise of anatomic boundaries while the latter comprised infections of the superficial surface of the wound and sub-dermal infection.

### Data analysis

Data were analyzed using SPSS 25.0. ORT and wound closure time were coded in minutes and measured as continuous variables. Other continuous variables concerned wound closure speed, the length of incision, the length of hospital stay and cost. 90-day readmission and wound complications were coded as dichotomous variables, one type of categorical variable. For continuous variables, the mean and range (± SD) were calculated. For categorical variables, the absolute number and frequency were presented. Continuous variables were evaluated for normal distribution using the Shapiro–Wilk test. All variables were normally distributed thus independent t tests were used. Categorical variables were assessed using Chi-square test. A *P* value of 0.05 was assigned as the threshold for clinical significance.

## Results

Results on the outcome measures of the two groups are summarized in Tables 2 and 3. ORT for the barbed suture group was 164.18 min, significantly shorter than 181.85 min for the conventional suture group (*P* < 0.05). Wound closure time in the study group was 8.12 min, compared with 25.36 min in the control group (*P* < 0.001). The lengths of incision between the two groups were statistically comparable (*P* = 0.086). Taking into consideration the incision length, the average suturing time in the study group was 0.65 cm/min, significantly shorter than 1.69 cm/min in the control group (*P* < 0.01). No statistically significant differences in length of hospital stay (LOS) between the two groups, 10.75 and 11.5 days for the study group and the control, respectively, were reported (*P* = 0.174). The suture material cost was higher in the barbed suture group compared with the conventional suture group: ¥883.5 ($138.24) vs. ¥441.67 ($69.11), whereas no statistical differences were observed between the two groups in terms of the total hospitalization expenses (*P* = 0.205). No differences were evidenced in 90-day readmission rates between the two groups with three cases (3.3%) and four cases (4.4%) for the barbed suture group and the conventional suture group, respectively (*P* = 0.232).

**Table 2:** Statistical analysis comparing barbed suture group and conventional suture group

	Barbed suture (N = 91)	Conventional suture (N = 90)	*t*	*P*
Operating room time (ORT) (min)	164.18 ± 46.50	181.85 ± 64.42	2.117	0.036
Wound closure time (min)	8.12 ± 4.75	25.36 ± 12.59	13.037	< 0.001
Length of incision (cm)	12.44 ± 7.19	15.89 ± 8.78	1.726	0.086
Average suturing time (cm/min)	0.65 ± 0.05	1.69 ± 0.25	37.635	< 0.001
Length of hospital stay (LOS) (day)	10.75 ± 2.68	11.5 ± 2.12	1.332	0.174
Suture material cost (¥)	883.5 ± 202.35	441.67 ± 61.43	19.83	< 0.001
Total hospitalization expenses (¥)	103,654 ± 14,653	108,713 ± 12,509	1.213	0.205

**Table 3:** Wound complications

	Barbed suture (N = 91)	Conventional suture (N = 90)	*P*
Wound dehiscence	0	3	
Wound infection	1	4	
Total	1	7	0.031
90-day readmission	3	4	0.232

In terms of complications, in the barbed suture group, none of patients were found with postoperative wound dehiscence (0%) and one patient developed wound infection (1.1%) (Table 3). In the conventional suture group, postoperative wound dehiscence and infection were found in three patients (3.3%) and four patients (4.4%), respectively.

## Discussion

As a critical component in the surgery, a safe and effective wound suture technique would make significant contributions to guaranteeing the success of the operation. In the past years, conventional knotting sutures were traditionally and dominantly applied in clinical practice [15]. But the procedure of tying the knots is gradually found to be time-consuming. Recently, the absorbable barbed sutures without knots began to emerge as a possible alternative and prevail in various surgical fields.

In the surgical area of orthopedics, while previous attempts of comparing the clinical outcomes between barbed sutures and conventional sutures were made in total knee arthroplasty and hip arthroplasty [16, 17], to the best of our knowledge, only three prior studies have investigated the employment of barbed sutures in spinal surgery [8, 9, 15]. They all found barbed sutures associated with shorter wound closure time or suture time but varied in the findings of hospital costs. These findings share similarities with those of the current study, though with differences observed.

In the present research, barbed sutures were related with significantly lower ORT, wound closure time and average wound closure time, and significantly lower wound complication rates (dehiscence and infection). However, no significant differences were found when compared with conventional sutures in terms of length of incision, length of hospital stay, readmission rates up to 90 days after the surgical procedure and costs.

Our results indicated significant lower ORT when incorporating barbed sutures, suggesting clinical efficiency of using barbed sutures in posterior long-segment lumbar surgery as overall ORT was measured as an efficiency outcome, consistent with previous findings [9, 12]. Additionally, our findings revealed that applying barded sutures cost significantly shorter time than the traditional sutures both in the entire wound closure procedure (17.24 min difference, *P* < 0.001) and wound closure for per centimeter of incision (1.04 min difference, *P* < . 001), i.e., average wound closure time, in line with earlier findings in and outside spinal surgery [5–13]. This is probably due to the need for interrupted knots in traditional sutures, which would extend operative and suturing time.

Aside from clinical outcomes, findings of postoperative complications were as follows: postoperative wound dehiscence occurred in three patients in conventional suture group, while none in barbed suture group, and four patients in conventional group were reported with wound infection while one in barbed suture group.

Lower wound dehiscence rate related to barbed sutures could be indicative of the reliable strength of the novel suture technique owing to evenly distributed tension of the whole suture. Research in other surgeries also reported lower probability of suture fracture and incision crack after two-way barbed suture closure [10].

Postoperative wound infection has been evidenced in more than 10% of spinal surgeries, resulting in patient discomfort, need for antibiotic treatment, prolonged hospitalization, and revision surgery [19–23]. Therefore, it has been a critical wound complication that deserves special attention in spinal surgeries. In earlier studies, no statistical differences in wound infection rates were reported between the barbed sutures and conventional sutures groups [9, 15]. However, our results yielded disparate outcomes. Applying barbed sutures has significantly lowered infection rates. Support from literature could be located. Operation time and the degree of incision stretch are high-risk independent factors for postoperative infection in spinal surgeries. The knots in traditional sutures may influence vascular supply and inflammatory reaction [15, 24, 25]. To further justify the different results from earlier studies, possible explanations could be the considerably larger incision length in posterior long-segment lumbar surgery compared to other spinal surgeries previously reported, which thereby may increase the risks of infection due to the multiple knots needed to be tied in traditional sutures.

No differences between the two groups were found in length of incision, length of hospital stays, 90 days readmission rates and costs.

Results were consistent with earlier research findings in indicating similar lengths of incision [8, 15] and lengths of hospital stays [9]. Though readmission rates were not a frequent outcome measure in prior studies, the non-significant differences were documented in Johnston’s retrospective study.

The non-significant differences in costs need special mentioning because they contradict with most of research findings that highlighted the cost-effective feature of barbed sutures [8, 9]. This may be largely due to the lower ORT since the ORT directly relates to operation cost in many hospitals. However, in the hospital. the present study was undertaken, higher ORT does not increase hospitalization expenses. In addition, even though the material cost was higher for the barbed sutures (¥883.5) than the conventional sutures (¥441.67), it accounted for only a small portion of the total hospitalization expenses, respectively (¥103,654 for barbed sutures and ¥108,713 for conventional sutures). Therefore, the use of two sutures did not lead to significant differences in total hospitalization costs.

## Conclusions

In this randomized controlled trial of patients undergoing posterior long-segment lumbar surgery, results indicated the knotless barbed suture technique outperformed the conventional suture in shortening operating room time, wound closure time and average wound closure time, and reducing wound complication rates. The two suturing techniques were comparable in terms of length of incision, length of hospital stay, readmission rates up to 90 days after the surgical procedure and hospitalization costs.

## Data Availability

The datasets generated during and/or analyzed during the current study are not publicly available because some results are yet to be published but are available from the corresponding author on reasonable request.

## References

[1] Piper K, Tomlinson S, Santangelo G (2017). Risk factors for wound complications following spine surgery. Surg Neurol Int.

[2] Manoucheri E, Einarsson JI (2013). The use of barbed suture in hysterectomy and myomectomy. Surg Technol Int.

[3] Nemecek E, Negrin L, Beran C, Nemecek R, Hollinsky C (2013). The application of the V-Loc closure device for gastrointestinal sutures: a preliminary study. Surg Endosc.

[4] Paul MD (2013). Barbed sutures in aesthetic plastic surgery: evolution of thought and process. Aesthet Surg J.

[5] Chan VW, Chan PK, Chiu KY (2017). Does barbed suture lower cost and improve outcome in total knee arthroplasty? A randomized controlled trial. J Arthroplasty.

[6] Zhang W, Xue D, Yin H (2016). Barbed versus traditional sutures for wound closure in knee arthroplasty: a systematic review and meta-analysis. Sci Rep.

[7] Eickmann T, Quane E (2010). Total knee arthroplasty closure with barbed sutures. J Knee Surg.

[8] Mansour A, Ballard R, Garg S (2013). The use of barbed sutures during scoliosis fusion wound closure: a quality improvement analysis. J Pediatr Orthop.

[9] Johnston SS, Chen BP, Tommaselli GA, Jain S, Pracyk JB (2020). Barbed and conventional sutures in spinal surgery patients: an economic and clinical outcomes comparison. J Wound Care.

[10] Meena S, Gangary S, Sharma P, Chowdhury B (2015). Barbed versus standard sutures in total knee arthroplasty: a meta-analysis. Eur J Orthop Surg Traumatol.

[11] Borzio RW, Pivec R, Kapadia BH (2016). Barbed sutures in total hip and knee arthroplasty: what is the evidence?. A Meta-Anal Int Orthop.

[12] Smith EL, DiSegna ST, Shukla PY, Matzkin EG (2014). Barbed versus traditional sutures: closure time, cost, and wound related outcomes in total joint arthroplasty. J Arthroplasty.

[13] Sutton N, Schmitz ND, Johnston SS (2018). Comparing outcomes between barbed and conventional sutures in patients undergoing knee or hip arthroplasty. J Comp Eff Res.

[14] Ingle NP, Cong H, King MW, King MW, Gupta BS, Guidoin R (2013). Barbed suture technology. Biotextiles as medical implants.

[15] Chen J, Xie C-L, Xuan J, Yan Y-Z, Dou H-C, Zheng Z-M, Chen Y, Chen X-B, Wang X-Y, Wu A-M (2018). A novel knotless barbed suture technique for traumatic thoracolumbar fracture in posterior surgery. World Neurosurg.

[16] Sah AP (2015). Is there an advantage to knotless barbed suture in TKA wound closure? A randomized trial in simultaneous bilateral TKAs. Clin Orthop Relat Res.

[17] Ting NT, Moric MM, Della Valle CJ, Levine BR (2012). Use of knotless suture for closure of total hip and knee arthroplasties: a prospective, randomized clinical trial. J Arthroplasty.

[18] Kim JH, Byun SW, Song JY (2016). Barbed versus conventional 2-layer continuous running sutures for laparoscopic vaginal cuff closure. Medicine.

[19] Blam OG, Vaccaro AR, Vanichkachorn JS, Albert TJ, Hilibrand AS, Minnich JM, Murphey SA (2003). Risk factors for surgical site infection in the patient with spinal injury. Spine (Phila Pa 1976).

[20] Weinstein MA, McCabe JP, Cammisa FP (2000). Postoperative spinal wound infection: a review of 2,391 consecutive index procedures. J Spinal Disord.

[21] Valentini LG, Casali C, Chatenoud L, Chiaffarino F, Uberti-Foppa C, Broggi G (2008). Surgical site infections after elective neurosurgery: a survey of 1747 patients. Neurosurgery.

[22] Olsen MA, Mayfield J, Lauryssen C, Polish LB, Jones M, Vest J, Fraser VJ (2003). Risk factors for surgical site infection in spinal surgery. J Neurosurg.

[23] Hellbusch LC, Helzer-Julin M, Doran SE (2008). Single-dose vs multiple-dose antibiotic prophylaxis in instrumented lumbar fusion-a prospective study. Surg Neurol.

[24] Yilmaz E, Tawfik T, O'Lynnger TM (2018). Wound closure after posterior multi-level lumbar spine surgery: an anatomical cadaver study and technical note. Cureus.

[25] Miyazaki S, Kakutani K, Maeno K (2016). Surgical debridement with retention of spinal instrumentation and long-term antimicrobial therapy for multidrug-resistant surgical site infections after spinal surgery: a case series. Int Orthop.

